# METTL3-regulated m6A modification impairs the decidualization of endometrial stromal cells by regulating YTHDF2-mediated degradation of FOXO1 mRNA in endometriosis-related infertility

**DOI:** 10.1186/s12958-023-01151-0

**Published:** 2023-10-27

**Authors:** Xiaoou Li, Jie Jin, Xuefeng Long, Ruiwen Weng, Wenqian Xiong, Jiaxin Liang, Junjun Liu, Jingwen Sun, Xueqin Cai, Ling Zhang, Yi Liu

**Affiliations:** Department of Obstetrics and Gynecology, Union Hospital, Tongji Medical College, Huazhong University of Science and Technology, Wuhan, 430022 Hubei China

**Keywords:** Decidualization, Endometriosis, Infertility, N6-methyladenosine, Methyltransferase-like 3, Forkhead box O1

## Abstract

**Background:**

Endometriosis-related infertility is a common worldwide reproductive health concern. Despite ongoing research, the causes of infertility remain unclear. Evidence suggests that epigenetic regulation is crucial in reproduction. However, the role of N6-methyladenosine (m6A) modification of RNA in endometriosis-related infertility requires further investigation.

**Methods:**

We examined the expression of m6A and methyltransferase-like 3 (METTL3) in endometrial samples taken from normal fertile women in the proliferative phase (the NP group) or the mid-secretory phase (the NS group) or from women with endometriosis-related infertility at the mid-secretory phase (the ES group). We treated primary endometrial stromal cells (ESCs) with medroxyprogesterone acetate and 8-Bromo-cyclic adenosine monophosphate for in vitro decidualization and detected the expression of m6A, METTL3, and decidual markers. We analyzed the expression of m6A, METTL3, and forkhead box O1 (FOXO1) in ESCs from normal fertile women (the ND group) or women with endometriosis-related infertility (the ED group). We also assessed the expression of m6A, METTL3, and decidual markers, as well as the embryo adhesion rate, upon *METTL3* overexpression or knockdown. Additionally, we investigated the role of METTL3 in embryo implantation in vivo by applying mice with endometriosis. Furthermore, we performed RNA stability assays, RNA immunoprecipitation (RIP), and methylated RIP assays to explore the mechanisms underlying the regulation of FOXO1 by METTL3-mediated m6A.

**Results:**

The expression of m6A and METTL3 was reduced only in the NS group; the NP and ES groups demonstrated increased m6A and METTL3 levels. m6A and METTL3 levels decreased in ESCs with prolonged decidual treatment. Compared to the ND group, m6A and METTL3 levels in the ED group increased after decidual treatment, whereas the expression of FOXO1 decreased. *METTL3* overexpression suppressed the expression of decidual markers and embryo implantation in vitro; *METTL3* knockdown exhibited the opposite effect. Inhibition of METTL3 promoted embryo implantation in vivo. Furthermore, we observed that METTL3-mediated m6A regulated the degradation of *FOXO1* mRNA through YTHDF2, a m6A binding protein.

**Conclusions:**

METTL3-regulated m6A promotes YTHDF2-mediated decay of *FOXO1* mRNA, thereby affecting cellular decidualization and embryo implantation. These findings provide novel insights into the development of therapies for women with endometriosis-related infertility.

**Supplementary Information:**

The online version contains supplementary material available at 10.1186/s12958-023-01151-0.

## Background

Infertility is a prevalent health issue affecting approximately 8–12% of couples worldwide [1, 2]. Endometriosis, a condition characterized by the presence of tissue similar to the lining of the uterus outside the uterus, is a multifactorial and systemic disease prevalent in 10% of women of reproductive age [3]. Approximately 50% of women with infertility suffer from endometriosis [4] and one third of women with endometriosis experience infertility [3]. Successful implantation of embryos in the uterus requires a well-functioning and synchronously developing endometrium, called the receptive endometrium, during the implantation window within the mid-secretory phase [5]. However, women with endometriosis frequently experience endometrial disorders [6] and lower rates of implantation and pregnancy than those with tubal infertility after in vitro fertilization (IVF) or intracytoplasmic sperm injection (ICSI) treatments [7, 8]. Decidualization, the process by which endometrial fibroblast-like stromal cells transform into specialized decidual cells, is crucial for establishing endometrial receptivity, providing a nutritional and immunosuppressive environment for embryo implantation [7]. Women with endometriosis-related infertility experience impaired decidualization, which contributes to endometrial defects [9–11]. Therefore, understanding the mechanisms involved in decidualization and endometrial receptivity is essential for the detection and treatment of endometriosis-related infertility.

N6-methyladenosine (m6A), one of the most prevalent RNA modifications, has dynamic and reversible regulatory features. The catalysis of m6A modifications is facilitated by a methyltransferase complex comprising two subcomplexes, methyltransferase-like 3 (METTL3) and 14 (METTL14), along with other components such as WT1 associated protein (WTAP), vir like m6A methyltransferase associated (VIRMA, also known as KIAA1429) [12]. Conversely, fat mass and obesity-associated protein (FTO) [13] and alkB homolog 5 (ALKBH5) [14] facilitate the reverse action. A balance between m6A methyltransferases and demethylases is involved in the dynamic regulation of m6A. The recognition of m6A modifications is attributed to reader proteins, including YTH domain-containing proteins (YTH N6-methyladenosine RNA binding protein C1/2 (YTHDC1/2) and F1/2/3 (YTHDF1/2/3)) and insulin-like growth factor 2 mRNA-binding protein 1/2/3 (IGF2BP1/2/3) [15]. Several studies have demonstrated the link between m6A modification and gametogenesis and fertility in both sexes [16, 17]. Additionally, m6A modifications are associated with the development of endometrium-related diseases such as endometrial cancer [18] and endometriosis [19, 20]. However, the precise role of m6A modifications in endometriosis-related infertility and endometrial decidualization remains unclear.

The objective of this study was to elucidate the functional role of m6A modification in cellular decidualization and to explore whether it is abnormal in women afflicted with endometriosis-related infertility. Additionally, we attempted to uncover the effect of METTL3 and m6A expression on the decidualization of endometrial stromal cells and the potential mechanisms involved, thereby offering a novel therapeutic approach for improving endometriosis-associated infertility.

## Materials and methods

### Patients and tissue collection

This study was approved by the local ethics committee of the Union Hospital, Tongji Medical College, Huazhong University of Science and Technology. Written informed consent was obtained from patients before the collection of human tissues, in accordance with the guidelines of the Declaration of Helsinki.

Normal control endometrial samples were obtained from patients without endometriosis who visited our hospital with tubal infertility. These samples included proliferative (*n* = 21) and mid-secretory (*n* = 21) phase endometria collected through curettage. Additionally, eutopic endometria in the mid-secretory phase (*n* = 14) were obtained from patients with stage III and IV ovarian endometriosis [21]. All the patients were premenopausal and had regular menstrual cycles. Menstrual cycle phases were determined based on their menstrual history and endometrial histology confirmed by an independent pathologist. None of the patients received hormonal treatment for at least three months prior.

### Cell culture and in vitro decidualization

Primary endometrial stromal cells (ESCs) were isolated from the samples collected from the proliferative stage of the menstrual cycle by Pipelle biopsy from fertile, regularly cycling women and endometriosis-associated infertile women under anesthesia as described previously [22]. The cells were routinely incubated in Dulbecco’s Modified Eagle’s Medium (DMEM/F12) without phenol red, containing 10% activated carbon-adsorbed serum (BasalMedia, Shanghai, China). When performing in vitro decidualization, cells were incubated in DMEM/F12 without phenol red containing 2% carbon-adsorbed serum with medroxyprogesterone acetate (MPA, 100 nM, MCE, HY-B0469S) and 8-Bromo-cyclic adenosine monophosphate (8-Br-cAMP, 0.5 mM, Selleck, S7857) added to the cellular supernatant. After 2–6 d, cellular decidualization was assessed by evaluating the expression of decidualization marker genes and cell morphology.

Human endometrial stromal cells (ThESCs) were purchased from the American Type Culture Collection (CRL-4003; ATCC) and cultured in the same medium and environment as ESCs. For METTL3 intervention assays, ThESCs were pretreated with a *METTL3*-overexpressing vector (Dianjun, Shanghai, China), siRNAs of METTL3 (Dianjun, Shanghai, China), or their own negative control group for 24 h and then treated with MPA and 8-Br-cAMP for 4 d. The siRNA sequences are listed in Table S2.

### RNA m6A quantitative assays

An EpiQuik m6A RNA Methylation Quantification Kit (Epigentek, NY, USA) was used to quantify the m6A levels in total RNAs. First, the RNAs were added to strip wells containing an RNA high-binding solution. According to the instructions, capture and detection antibody solutions were then added separately to the wells at appropriate dilutions. Finally, m6A levels were measured colorimetrically by measuring the absorbance at 450 nm using a microplate reader. Data were calculated using relative quantification.

### RNA isolation and quantitative real-time polymerase chain reaction (qRT-PCR)

Total RNAs were extracted from cells using TRIzol (Vazyme, Nanjing, China) following the manufacturer’s instructions. cDNA was synthesized and quantified using a HiScript III 1st Strand cDNA Synthesis Kit (Vazyme, Nanjing, China). qRT-PCR was conducted with 2 × TSINGKE® Master qPCR Mix (SYBR Green I with UDG; Tsingke, Beijing, China). All data were analyzed using the 2^−ΔΔCt^ method. The primer sequences used are presented in Table S1.

### Western blot analysis

Radioimmunoprecipitation assay buffer (Beyotime) was used to extract proteins, which were then quantified using a BCA protein assay kit (Beyotime). Equal amounts of protein (30 μg) were resolved using sodium dodecyl sulfate–polyacrylamide gel electrophoresis and transferred onto polyvinyl difluoride membranes (Millipore, MA, USA). After blocking with 5% skimmed milk in Tris-buffered saline containing 0.1% Tween-20 (TBST) for 1 h, the membranes were incubated with primary antibodies against METTL3 (1:1000, Abcam, MA, USA), forkhead box O1 (FOXO1, 1:1000, CST, MA, USA), β-actin (1:200000, Proteintech, Wuhan, China), and glyceraldehyde-3-phosphate dehydrogenase (GAPDH; 1:200000, Proteintech, Wuhan, China) overnight at 4 °C. After washing, the membranes were incubated with anti-rabbit antibody (1:5000, Proteintech, Wuhan, China) at 25 °C for 1 h. After washing, the membranes were visualized using a detection system, followed by incubation with enhanced ECL detection reagent (Biology, Wuhan, China). The gray values of the protein bands were analyzed using Image J software.

### Immunohistochemical (IHC) staining

All the tissues were immediately placed in 4% buffered formalin for IHC staining. Paraffin embedding, sectioning, and IHC staining were performed by Biosciences Biotechnology Co., Ltd. (Wuhan, China). IHC staining of paraffin sections was performed using primary antibodies against METTL3 (1:400; Abcam, Cambridge, USA) and FOXO1 (1:1000; Cell Signaling Technology, Danvers, MA, USA). Finally, Image-Pro Plus software 6.0 was used to analyze the data.

### Immunofluorescence (IF) staining

Primary stromal cells were fixed with 4% paraformaldehyde for 30 min at 25 °C and then permeabilized with phosphate buffer saline (PBS) containing 0.1% TritonX-100 for 10 min at 25 °C. Non-specific sites were blocked with 1% bovine serum albumin in PBS for 1 h at 37 °C. Endogenous proteins were stained with primary antibodies against METTL3 (1:400, Abcam, MA, USA) and FOXO1 (1:1000, CST, MA, USA) for 1 h at 25 °C. Fluorescence-conjugated secondary antibodies (1:4000; Proteintech) were used to visualize the signals. The nuclei were stained with 4’,6-diamidino-2phenylindole dihydrochloride for 10 min. Finally, images were obtained by fluorescence confocal microscopy and processed using Image Pro Plus 6.0 software.

### Enzyme-linked immunosorbent assay

After in vitro cellular decidualization, cell culture supernatants were harvested and centrifuged to remove cell debris. A commercially available prolactin (PRL) enzyme-linked immunosorbent assay kit (ruixinbio, Quanzhou, China, RX106036H) and insulin-like growth factor-binding protein 1 (IGFBP1) enzyme-linked immunosorbent assay kit (ruixinbio, Quanzhou, China, RX104919H) were used to detect PRL and IGFBP1 levels, respectively, in the collected supernatants. The samples were assayed in duplicate, and the concentrations were expressed as mIU/mL or ng/mL of the cell supernatant.

### RNA m6A dot blot assays

Total RNAs were spotted onto N^+^ nylon membranes (GE Healthcare, MD, USA). After ultraviolet cross-linking, the membranes were blocked with 5% fat-free milk in TBST for 1 h and then incubated with an anti-m6A antibody (1:1000, Proteintech, Wuhan, China) overnight at 4 °C. After washing, the membranes were incubated with an anti-mouse antibody (1:5000, Proteintech, Wuhan, China) for 1 h at 25 °C. After further washing, the membranes were incubated with enhanced ECL detection reagent (Biology, Wuhan, China) and visualized using a detection system. After washing, the membranes were stained with 0.2% methylene blue as a control.

### In vitro embryo implantation assays

HTR-8/SVneo cells (from an immortalized cell line derived from first-trimester villous explants) were co-cultured with a confluent monolayer of ThESCs to simulate embryo attachment [23]. First, a single-cell suspension of HTR8 cells was placed in a low-adhesion 96-well plate. Multicellular spheroids of HTR8 cells were induced after 72 h of culture and 70–100 μm diameter multicellular spheroids were sieved through filter sieves. Simultaneously, HTR8 spheroids were transferred onto a confluent monolayer of ThESCs, which were treated accordingly in advance. After incubation at 37 °C for 12 h, cells were washed with PBS to remove the unattached spheroids. The attached spheroids were counted under a light microscope, and the adhesion rate was expressed as a percentage of the total number of HTR8 spheroids added to the ThESC monolayer.

### Animal experiments

C57BL/6 mice (*n* = 57) were purchased from Hubei Beiente Biotechnology Co., Ltd. (Wuhan, China). Five female mice were subjected to a normal pregnancy assay. Uterine tissues of 14 female donor mice were cut up and injected into the abdominal cavity of 28 female recipient mice. After 21 d, 10 male C57 mice were mated with the recipient mice, and the next day, when vaginal plugs were observed, was regarded as day 1. Eight recipient mice were euthanized by cervical dislocation after deep pentobarbital anesthesia on day 8, and the number of blastocysts in the uterus was counted. At the night of day 3, 10 μL of STM2457, a METTL3 inhibitor (Sellcek, S9870, 10 μM) and 10 μL dimethyl sulfoxide (DMSO) were individually injected into the uterine horns of 20 recipient mice. The mice were euthanized on day 8, and the number of blastocysts in the uterus was counted. The uterine tissues were collected and fixed in 4% (w/v) paraformaldehyde for histological and IHC analyses. All animal experiments were approved by the Ethics Committee of the Animal Center of the Tongji Medical College (approval number 3332).

### RNA immunoprecipitation PCR (RIP-PCR)

An RNA immunoprecipitation kit (Bersinbio RIP Kit, Guangzhou, China) was used to perform the RIP assays. The cells were UV-irradiated and lysed with lysis buffer according to the manufacturer’s instructions. Immunoprecipitation of endogenous factors was performed using a primary antibody overnight at 4 °C. Protein A/G beads were then added to capture the primary antibody. After washing, proteinase K was added to the immunoprecipitated complex to remove excess proteins. RNAs were extracted using TRIzol reagent and quantified by qRT-PCR using primers for *FOXO1*. The data were normalized to input or %IgG of input.

### Methylated RIP-PCR (MeRIP-PCR)

A methylated RNA immunoprecipitation kit (Bersinbio RIP Kit, Guangzhou, China) was used to perform the MeRIP assays. RNAs were isolated from cells and fragmented by ultrasonication for 1.5 min. An anti-m6A antibody was used for immunoprecipitation. Protein A/G beads were then added to capture the anti-m6A antibody. After several washes, proteinase K was added to the immunoprecipitated complex to remove excess proteins. Finally, RNAs were extracted using TRIzol reagent and quantified by qRT-PCR using primers for *FOXO1*. The data were normalized to the input or %IgG of input.

### RNA stability assays

ThESCs were pretreated with a *METTL3*-overexpressing vector (Dianjun, Shanghai, China) and its control group (or with the wild-type [ovM3-WT, Dianjun, Shanghai, China], mutated *METTL3*-overexpressing vector [ovM3-MUT, D395A and W398A, Dianjun, Shanghai, China], and their control group) for 24 h and then treated with MPA and 8-Br-cAMP for 4 d. Next, actinomycin D (Act D, Selleck, S8964) was added to the culture media with a final concentration of 2 μg/mL and cells were collected at 2, 4, and 6 h time points following actinomycin D addition. Finally, RNAs were extracted using TRIzol reagent and quantified by qRT-PCR using primers for *FOXO1*. The mutant of the *METTL3*-overexpressing vector was constructed with disordered enzymatic activity, as described previously [24].

### Statistical analysis

GraphPad Prism 7 was used for the statistical analyses. All data were presented as the mean ± standard error of the mean. All experiments were repeated in triplicate or quadruplicate. For data variables with a normal distribution, a Student’s* t* test was used to analyze differences between the two groups and a one-way analysis of variance was performed to analyze differences between multiple groups. For non-normally distributed data, the Mann–Whitney test was used for two groups, while the Kruskal–Wallis test was used for multiple groups. Statistical significance was defined as *P* < 0.05.

## Results

### m6A and METTL3 are downregulated in mid-secretory phase endometria from normal fertile women

**Fig. 1:**
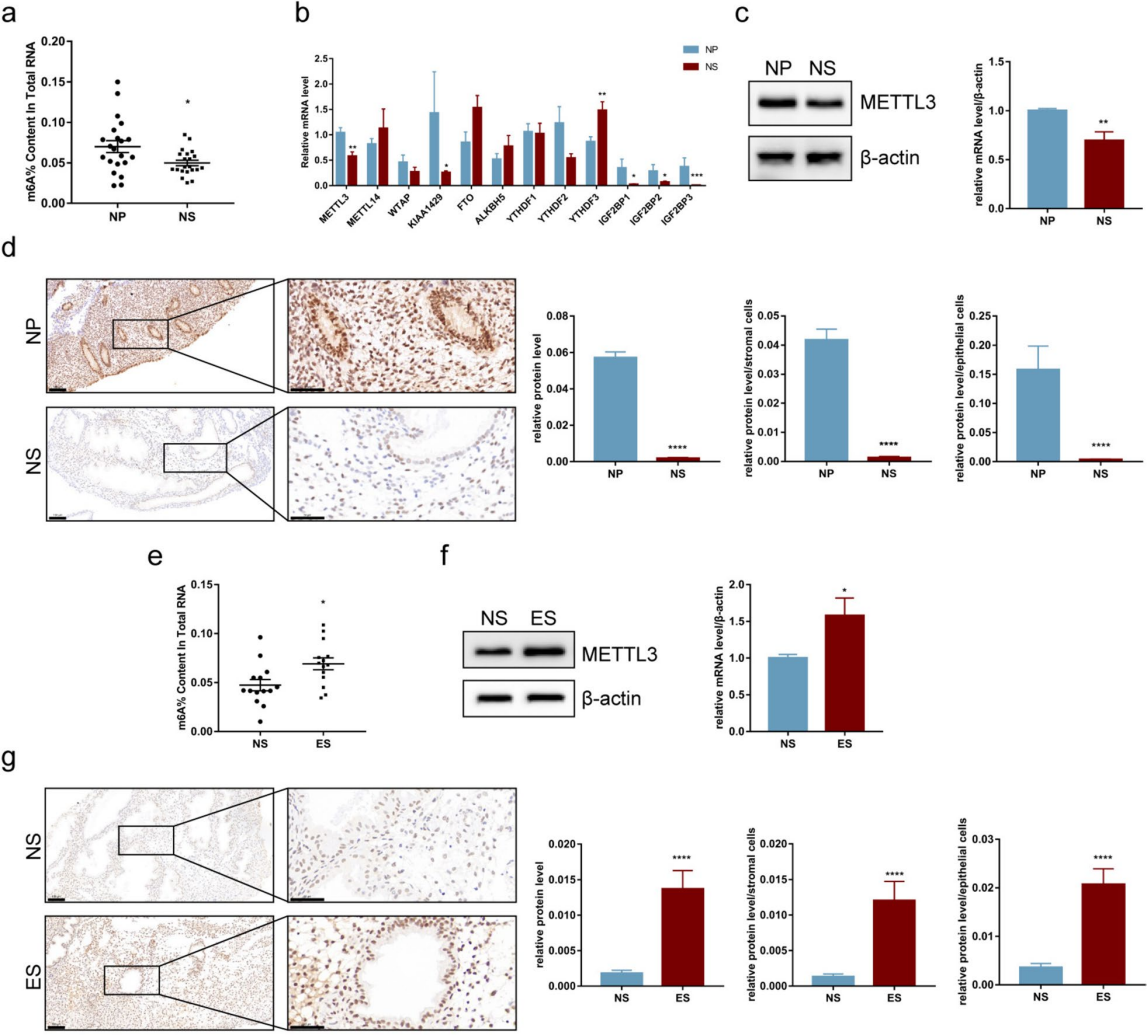
Expression of N6-methyladenosine (m6A) and methyltransferase-like 3 (METTL3) in different endometrial samples. m6A and METTL3 levels were downregulated in the mid-secretory endometria of normal fertile women (the NS group) without endometriosis, whereas it was upregulated during the same phase in the endometria of women with endometriosis-related infertility (the ES group). **a** m6A levels in the endometria of normal fertile women in the proliferative phase (the NP group) (*n* = 21) and the NS group (*n* = 21). **b** mRNA levels of m6A-associated genes in the tissues detected by qRT-PCR. **c** Protein levels of METTL3 in sampled tissues detected by western blot analysis. **d** Protein levels of METTL3 in sampled tissues detected by immunohistochemical (IHC) staining (scale bar = 100 μm or scale bar = 50 μm). **e** m6A levels in the NS (*n* = 14) and ES (*n* = 14) groups. **f** Protein levels of METTL3 in sampled tissues detected by western blot analysis. **g** Protein levels of METTL3 in sampled tissues detected by IHC staining (scale bar = 100 μm or scale bar = 50 μm). All experiments were repeated in triplicate or quadruplicate. The blots in this figure are cropped (please refer to supplementary files for details). Data with error bars are presented to indicate the mean ± standard error of the mean (SEM) values. *P < 0.05, **P < 0.01, ***P < 0.001, ****P < 0.0001

To investigate the role of m6A modification in endometrial receptivity, we collected endometria from fertile women without endometriosis during both the proliferative phase (the NP group) and the mid-secretory phase (the NS group) and measured m6A levels using a colorimetric method. The results show that m6A levels in the NS group were downregulated compared to those in the NP group (Fig. 1a).

As previously stated, m6A is a dynamic RNA modification regulated by methyltransferases and demethylases. METTL3 and METTL14, core components of the m6A methyltransferase complex, are involved in the formation of m6A [12], whereas the demethylases FTO [13] and ALKBH5 [14] are involved in demethylation. Furthermore, emerging studies have proposed that m6A readers such as YTHDFs and IGF2BPs perform the real function of m6A by combining it with m6A tags [15]. Therefore, we measured the expression of these m6A-associated genes in the NP and NS samples using qRT-PCR to determine the key molecules involved in m6A downregulation in the NS group. The results show that *METTL3* and *KIAA1429* were downregulated in the NS group than in the NP group; however, no difference in the expression of *METTL14*, *WTAP*, *FTO*, and *ALKBH5* was observed between the NP and NS groups (Fig. 1b). In addition, *YTHDF3* expression was upregulated in the NS group compared to that in the NP group; however, no difference in *YTHDF1* and *YTHDF2* expression was observed between the NP and NS groups (Fig. 1b). Notably, the mRNA levels of all *IGF2BPs* were downregulated in the NS group compared with those in the NP group (Fig. 1b). These findings suggest an important role of m6A in endometrial receptivity.

As a catalytic factor in the methyltransferase complex, METTL3 was chosen to further investigate the function of m6A in endometrial receptivity. Western blot analysis and IHC staining were performed for further confirmation. The results showed that METTL3 was downregulated in the NS group than in the NP group (Fig. 1c,d), similar to the qRT-PCR results. In addition, we observed that METTL3 was expressed in the nuclei of epithelial and stromal cells and that METTL3 expression in both epithelial and stromal cells was downregulated in the NS group than in the NP group (Fig. 1d). Taken together, these results suggest that the downregulation of m6A and METTL3 in the mid-secretory phase may have a positive effect on the establishment of endometrial receptivity during the implantation window.

### m6A and METTL3 are upregulated in the mid-secretory phase of endometria from women with endometriosis-related infertility

Women with endometriosis often develop infertility, which is closely associated with defective endometrial receptivity [9–11]. To investigate the role of m6A and METTL3 in patients with endometriosis-related infertility, we collected mid-secretory endometrial samples from women with endometriosis-related infertility (the ES group). As expected, m6A levels in the ES group were upregulated compared to those in the NS group (Fig. 1e). In addition, METTL3 expression, as determined by western blot analysis and IHC staining, was upregulated in the ES group than in the NS group, consistent with m6A upregulation (Fig. 1f,g). Furthermore, we observed that METTL3 was upregulated in epithelial and stromal cells in the ES group than in the NS group (Fig. 1g). Taken together, these results suggest that the upregulation of METTL3 and m6A probably accounts for the impaired endometrial receptivity in women with endometriosis-related infertility.

### Reduction of m6A and METTL3 contributes to the decidualization of primary endometrial stromal cells

**Fig. 2:**
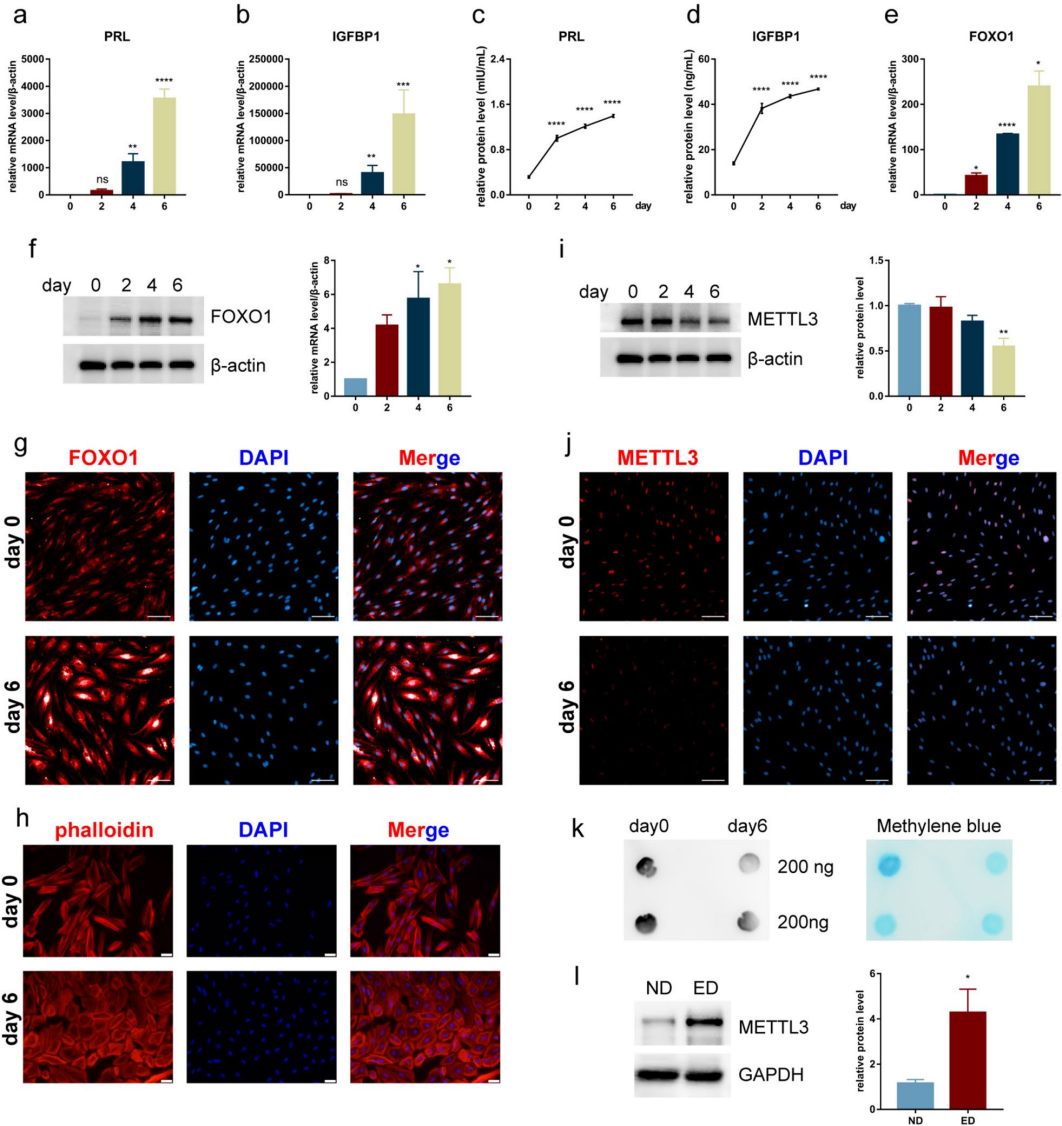
METTL3 expression was downregulated during the decidualization of primary endometrial stromal cells (ESCs) in vitro. These cells were treated with medroxyprogesterone acetate (MPA) and 8-Bromo-cyclic adenosine monophosphate (8-Br-cAMP) for the decidual treatment for 0, 2, 4, and 6 d. The mRNA (**a**, **b**) and protein (**c**, **d**) levels of prolactin (PRL) and insulin-like growth factor-binding protein 1 (IGFBP1) were detected using qRT-PCR and western blot analysis, respectively. **e**, **f** mRNA and protein levels of forkhead box O1 (FOXO1) were detected by qRT-PCR and western blot analysis, respectively. **g** Immunofluorescence (IF) staining was performed to detect FOXO1 levels (scale bar = 100 μm). **h** IF staining was performed to detect morphological changes in cells after decidual treatment (scale bar = 50 μm). **i**, **j** Protein levels of METTL3 were detected by western blot analysis and IF staining (scale bar = 100 μm). **k** Dot blot assays were performed to detect m6A changes in cells after decidual treatment. Methylene blue staining was used as a control. **l** Protein levels of METTL3 in ESCs derived from normal (ND) and endometriosis (ED) groups, both of which received decidual treatments. All experiments were repeated in triplicate or quadruplicate. The blots in this figure are cropped (please refer to supplementary files for details). Data with error bars are presented as the mean ± SEM values. **P < 0.01, ***P < 0.001, ****P < 0.0001

Decidualization of endometrial stromal cells is a crucial step in establishing endometrial receptivity [25]. Previous results indicate the potential role of m6A and METTL3 in establishing endometrial receptivity; therefore, we explored the function of METTL3 in the decidual process of stromal cells. First, we established in vitro decidualization of ESCs using MPA and 8-Br-cAMP treatment and examined the expression levels of decidual markers at different time points. The results showed that the mRNA and protein levels of PRL and IGFBP1 were increased over time, with the highest expression on day 6 (Fig. 2a-d). In addition, the mRNA and protein levels of FOXO1, which regulates the transcription of PRL and IGFBP1, increased over time (Fig. 2e,f). Furthermore, IF staining showed that the FOXO1 expression increased on day 6 (Fig. 2g, Supplementary Figure S1a). Additionally, compared to ESCs on day 0, cellular morphology on day 6 was more rounded (Fig. 2h). These results suggest successful establishment of in vitro decidualization. Next, we measured METTL3 and m6A levels after decidual treatment and found that METTL3 expression decreased over time, with the lowest expression observed on day 6 (Fig. 2i). IF staining also showed lower METTL3 expression on day 6 than on day 0 (Fig. 2j, Supplementary Figure S1b). In addition, the level of m6A on day 6 was lower than that on day 0 (Fig. 2k). These results suggest that decreased m6A and METTL3 levels contribute to the decidualization of endometrial stromal cells.

We then extracted ESCs derived from women with endometriosis-related infertility and performed the same in vitro decidualization assay for six days (the ED group). Compared with those of normal ESCs treated with in vitro decidualization (the ND group), m6A and METTL3 levels in the ED group were significantly increased (Fig. 2l, Supplementary Figure S1c), while the decidual marker FOXO1 was decreased (Supplementary Figure S1d). Taken together, these results suggest that the reduction in METTL3 and m6A levels contributes to the decidual process of endometrial stromal cells. Therefore, when m6A and METTL3 levels are increased in stromal cells derived from women with endometriosis-related infertility, cellular decidualization is disrupted, resulting in defective endometrial receptivity.

### METTL3 is involved in regulating the decidualization of endometrial stromal cells and embryo implantation

**Fig. 3:**
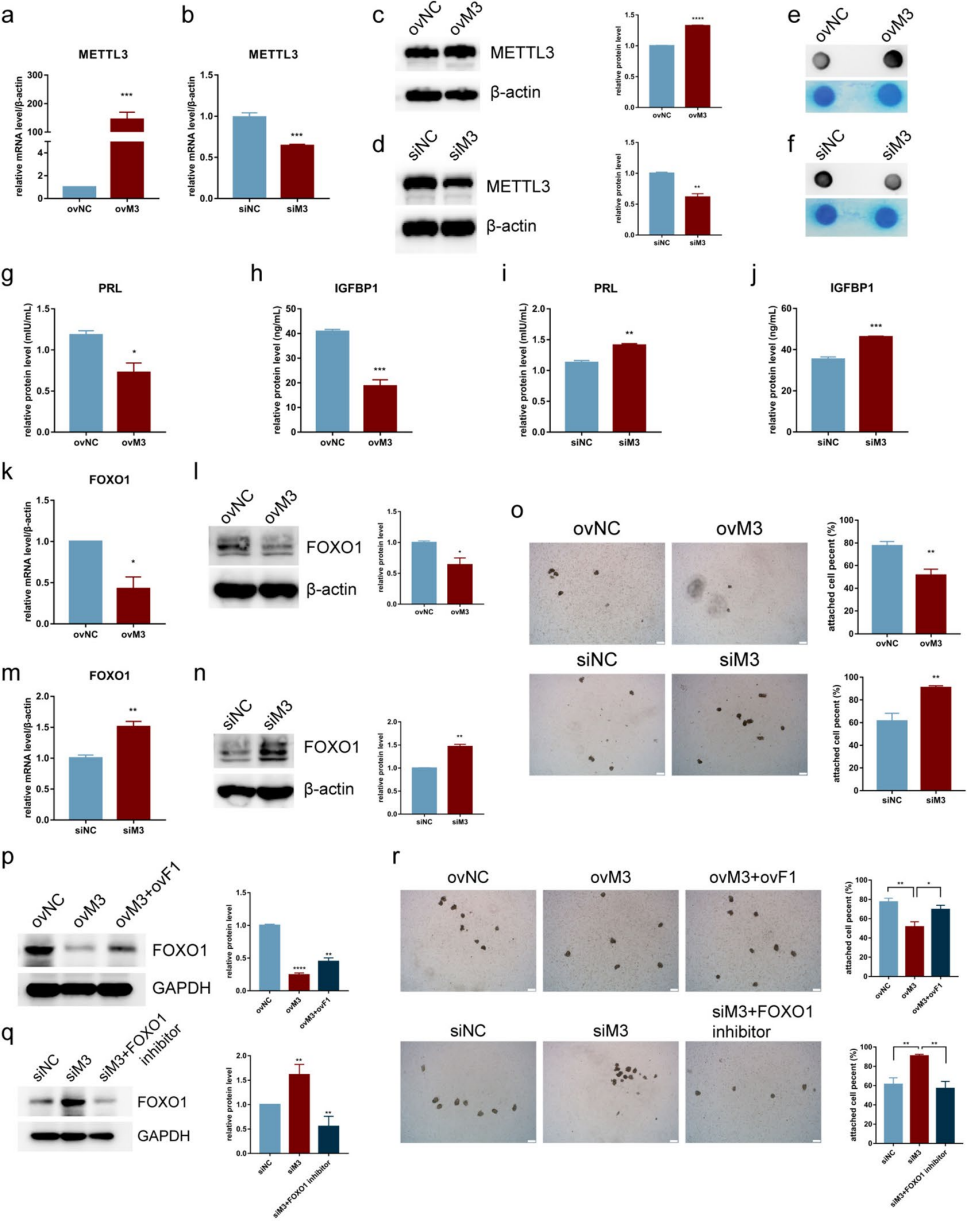
METTL3 was involved in regulating cellular decidualization and embryo implantation. **a**, **b** Overexpression and knockdown (ovM3, siM3) efficacy of METTL3 at the mRNA level in human endometrial stromal cells (ThESCs). **c**, **d** Overexpression and knockdown efficacy of METTL3 at the protein level in ThESCs. **e**, **f** Dot blot assays were used to detect m6A changes upon METTL3 overexpression or knockdown in ThESCs. Methylene blue staining was performed as a control. **g**-**j** Protein levels of PRL and IGFBP1 upon METTL3 overexpression or knockdown in ThESCs. **k**, **l** mRNA and protein levels of *FOXO1* upon METTL3 overexpression in ThESCs. **m**, **n** mRNA and protein levels of FOXO1 upon METTL3 knockdown in ThESCs. **o** Cell percentage of HTR-8/SVneo trophoblast cell line spheroids attached to ThESCs following METTL3 overexpression or knockdown were evaluated (scale bar = 200 μm). **p** Protein level of FOXO1 reduced by METTL3 overexpression was rescued by FOXO1 overexpression. **q** Protein level of FOXO1 increased by METTL3 knockdown was decreased by a FOXO1 inhibitor. **r** Cell percentage of HTR-8/SVneo spheroids attached to ThESCs transfected with the negative control and *METTL3*-overexpressing vector were evaluated in the absence or presence of the *FOXO1*-overexpressing vector. The cell percentage of HTR-8/SVneo spheroids attached to ThESCs transfected with the negative control and siRNA of *METTL3* were evaluated in the absence or presence of a FOXO1 inhibitor (scale bar = 200 μm). ThESCs were treated with METTL3 intervention for 24 h and then subjected to decidual treatment with MPA + 8-br-cAMP for 4 d. All experiments were repeated in triplicate or quadruplicate. The blots in this figure are cropped (please refer to supplementary files for details). Data with error bars are presented to indicate the mean ± SEM values. *P < 0.05, **P < 0.01, ***P < 0.001

To further confirm the correlation between METTL3/m6A and cellular decidualization, we transfected a *METTL3*-overexpressing vector or siRNAs of *METTL3* into ThESCs and then performed in vitro decidualization. *METTL3* was effectively overexpressed and silenced at the mRNA and protein levels (Fig. 3a-d). As expected, *METTL3* overexpression increased m6A levels compared to the negative control (Fig. 3e), whereas *METTL3* knockdown decreased m6A levels (Fig. 3f), suggesting that METTL3 regulates m6A levels in decidualizing stromal cells.

Next, we investigated the effect of changes in METTL3 expression on cellular decidualization. The results showed that *METTL3* overexpression decreased the mRNA and protein levels of PRL and IGFBP1 (Fig. 3g,h, Supplementary Figure S1e,f), whereas METTL3 knockdown increased the mRNA and protein levels of PRL and IGFBP1 (Fig. 3i,j, Supplementary Figure S1g,h). In addition, FOXO1 expression decreased at the mRNA and protein levels when *METTL3* was overexpressed (Fig. 3k,l), whereas FOXO1 expression increased when *METTL3* was silenced (Fig. 3m,n). These results suggest that METTL3 is involved in regulating the decidualization of stromal cells.

We then investigated whether alterations in METTL3 expression in stromal cells affect embryo implantation using in vitro embryo implantation assays. The results showed that *METTL3* overexpression in ThESCs reduced the adhesion rate of HTR8 spheroids from the HTR-8/SVneo trophoblast cell line, whereas METTL3 knockdown increased this rate (Fig. 3o). We further examined whether changes in METTL3 expression affect embryo implantation by regulating FOXO1 expression. The results showed that *FOXO1* overexpression rescued the reduced FOXO1 levels induced by *METTL3* overexpression (Fig. 3p); however, treatment with a FOXO1 inhibitor reduced the increased FOXO1 levels induced by METTL3 knockdown (Fig. 3q). Additionally, the overexpression of *FOXO1* restored the adhesion rate of HTR8 spheroids reduced by *METTL3* overexpression; however, treatment with a FOXO1 inhibitor reduced the adhesion rate increased by METTL3 knockdown (Fig. 3r). Overall, we demonstrate that METTL3 is involved in cellular decidualization by modulating FOXO1 expression, thereby affecting embryo implantation in vitro.

**Fig. 4:**
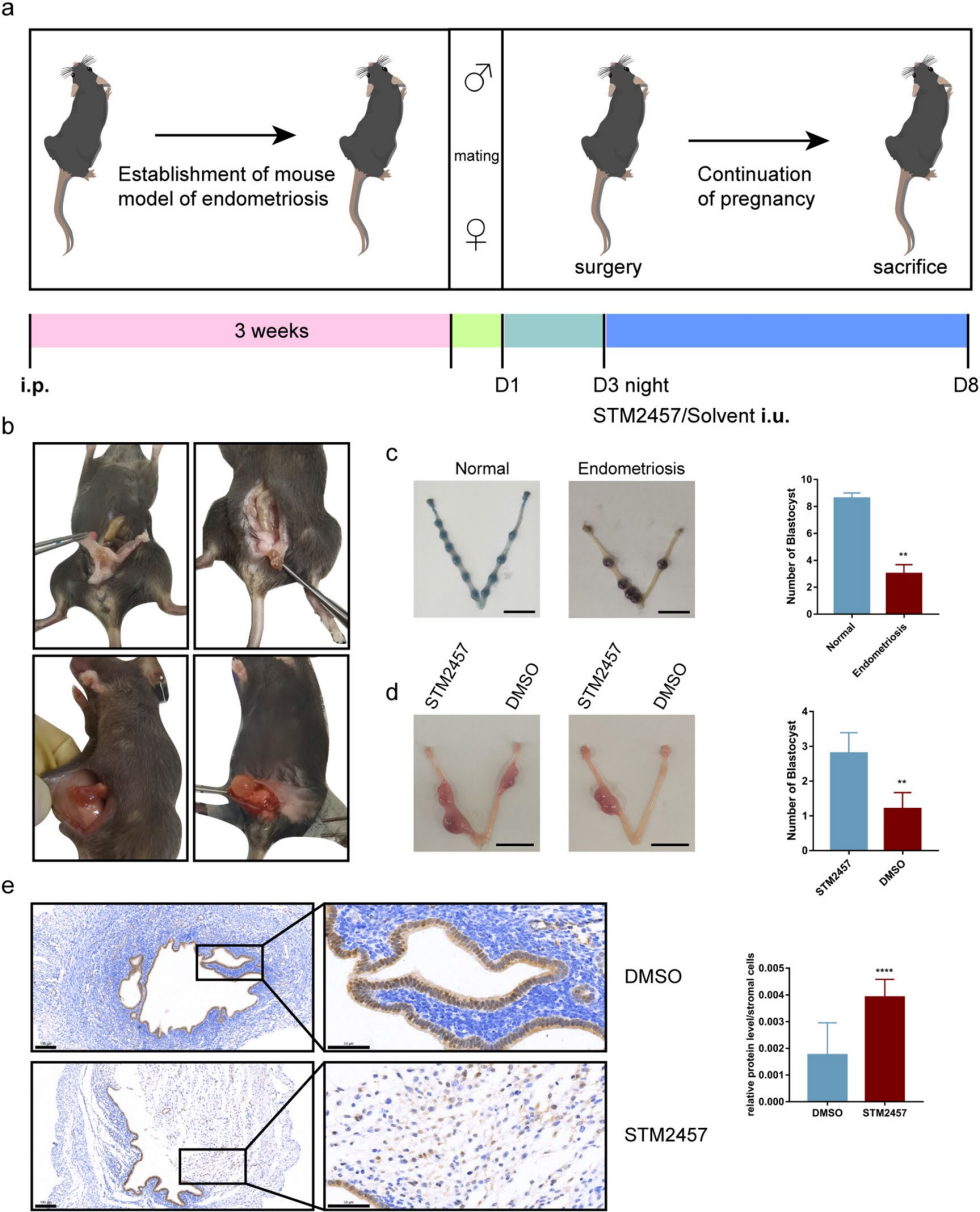
Changes in METTL3 expression were involved in embryo implantation by regulating cellular decidualization in vivo. **a** Flowchart of animal experiments. **b** Mouse models of endometriosis were established and the ectopic lesions are shown. **c** Number of blastocysts in normal pregnant mice (*n* = 5) and mice with endometriosis (*n* = 8) was counted (the left panel, scale bar = 1 cm). **d** Either the METTL3 inhibitor (STM2457) or the control solvent dimethyl sulfoxide (DMSO) were injected into the horn of the uterus on either side of the mice with endometriosis (*n* = 10). The number of blastocysts per side of the uterus was counted (the left panel, scale bar = 1 cm). **e** Protein levels of FOXO1 on the DMSO-injected and STM2457-injected sides of the mouse uterus were detected by IHC staining on day 8 (scale bar = 100 μm or scale bar = 50 μm). All experiments were repeated in triplicate or quadruplicate. Data with error bars are presented to indicate the mean ± SEM values. ***P < 0.001, ****P < 0.0001

For further confirmation, mouse models of endometriosis were established for in-depth in vivo investigation, as shown in Fig. 4a. Figure 4b shows the ectopic lesions of model mice with endometriosis. After mating, the day the virginal plugs were observed was regarded as day 1 (D1), and the mice were euthanized on day 8 (D8). The result showed that the number of blastocysts implanted in normal pregnant mice was significantly higher than that implanted in mice with endometriosis (Fig. 4c). Next, we injected an METTL3 inhibitor (STM2457) and control solvent (DMSO) individually into the horn of the uterus on opposite sides of the mice with endometriosis. The number of blastocysts implanted on the side injected with STM2457 was higher than that implanted on the side injected with DMSO (Fig. 4d). In addition, IHC staining showed that FOXO1 expression in stromal cells on the STM2457-injected side was higher than that on the DMSO-injected side (Fig. 4e); however, no difference in FOXO1 expression was observed in epithelial cells (Supplementary Figure S2). Taken together, these findings demonstrate that increased METTL3 expression impairs the decidualization of endometrial stromal cells and thus affects embryo implantation, which might largely contribute to endometriosis-related infertility.

### METTL3-mediated m6A promotes the degradation of FOXO1 mRNA in a YTHDF2-dependent manner

**Fig. 5:**
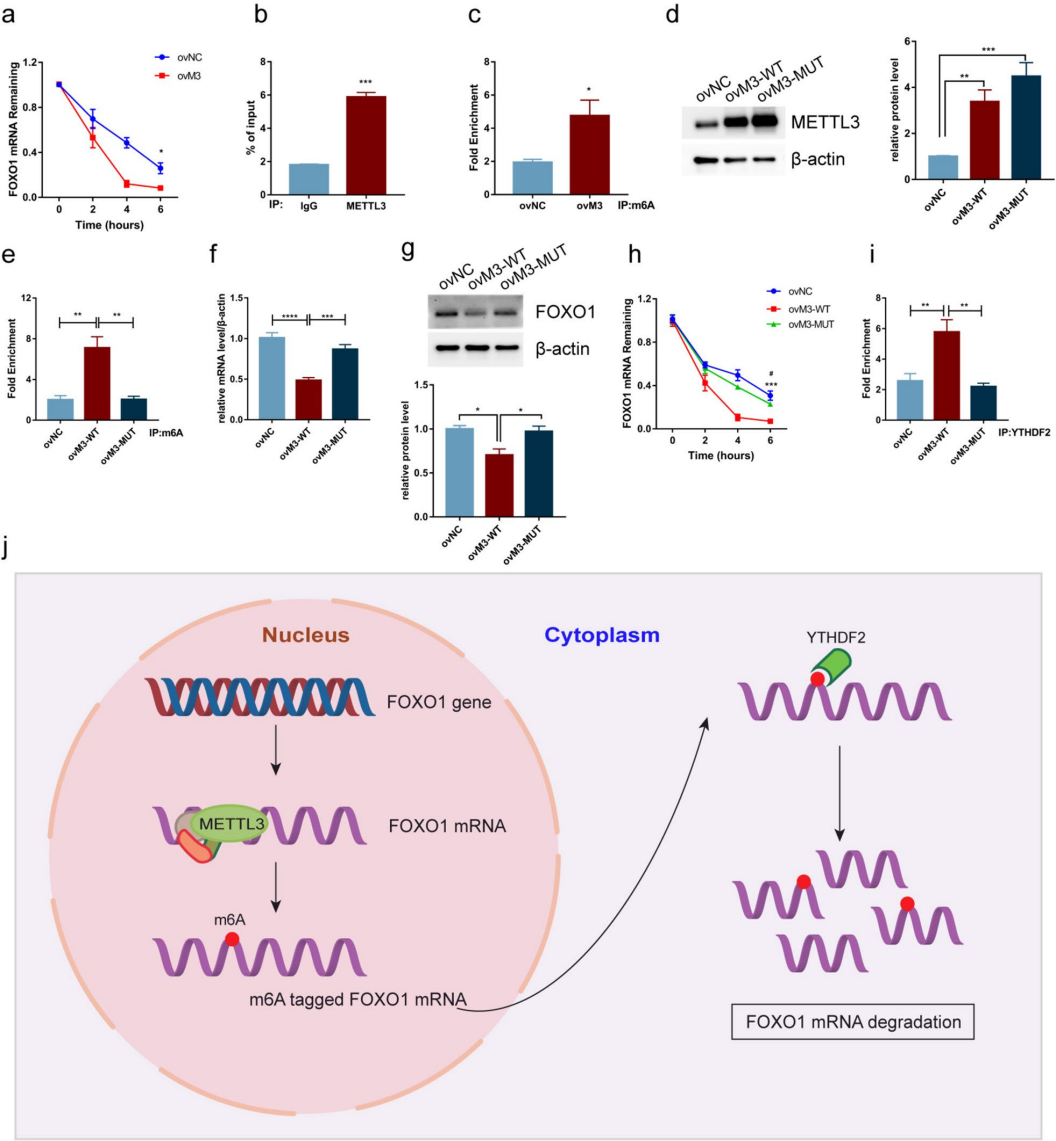
METTL3-mediated m6A regulated the degradation of *FOXO1* mRNA in a YTHDF2-dependent manner. **a** The curve and statistical analysis of the *FOXO1* mRNA decay slope in the negative or *METTL3*-overexpressing ThESCs after transcriptional inhibition. **b** RNA immunoprecipitation-PCR (RIP-PCR) assays showing an enrichment of *FOXO1* bound to METTL3 in ThESCs. **c** Methylated RNA immunoprecipitation-PCR (MeRIP-PCR) assays showing an enrichment of *FOXO1* with m6A in *METTL3*-overexpressing ThESCs. **d** Protein levels of METTL3 in the wild *METTL3*-overexpressing (ovM3-WT) and mutated *METTL3*-overexpressing (ovM3-MUT) ThESCs were analyzed by western blotting. **e** Methylation of *FOXO1* mRNA in the ovM3-WT and ovM3-MUT ThESCs were analyzed by MeRIP-PCR. **f** mRNA levels of *FOXO1* in the ovM3-WT and ovM3-MUT ThESCs were analyzed by qRT-PCR. **g** Protein levels of FOXO1 in the ovM3-WT and ovM3-MUT ThESCs were analyzed by western blotting. **h** Curve and statistical analysis of the *FOXO1* mRNA decay slope in the ovM3-WT and ovM3-MUT ThESCs after transcriptional inhibition. **i** Enrichment of *FOXO1* mRNA bound to YTHDF2 in the ovM3-WT and ovM3-MUT ThESCs were analyzed by RIP-PCR. **j** Model of a pattern of METTL3-mediated m6A in regulating the decidualization of endometrial stromal cells: METTL3 increases the m6A level of *FOXO1* mRNA, thus promoting the binding of YTHDF2 and enhancing the degradation of *FOXO1* mRNA, contributing to the defective decidualization of endometrial stromal cells in endometriosis. All experiments were repeated in triplicate or quadruplicate. The blots in this figure are cropped (please refer to supplementary files for details). Data with error bars are presented to indicate the mean ± SEM values. ***P < 0.001, ****P < 0.0001

We then investigated the potential mechanisms by which METTL3 regulates FOXO1 expression during the cellular decidualization process. Various studies have demonstrated that m6A is crucial in several RNA processes, including RNA transcription, splicing, stability, and translation [26–29], among which RNA stability and translation are the most widely studied. Based on previous results, we hypothesized that METTL3 regulates the stability of *FOXO1* mRNA in an m6A-dependent manner, thus affecting cellular decidualization. To test this hypothesis, *METTL3*-overexpressing cells with decidual treatment were treated with the transcriptional inhibitor Act D to determine the stability of *FOXO1* mRNA. The results showed a lower stability of *FOXO1* mRNA in *METTL3*-overexpressing cells (Fig. 5a), suggesting that *METTL3* overexpression promotes the decay of *FOXO1* mRNA during the decidual process. We then performed RNA immunoprecipitation on ThESCs using an anti-METTL3 antibody. The results showed that METTL3 coprecipitated with *FOXO1* (Fig. 5b). MeRIP-PCR using an anti-m6A antibody was then performed to test whether METTL3 regulates FOXO1 expression in an m6A-dependent manner. We observed that the level of *FOXO1* modified by m6A was elevated in *METTL3*-overexpressing cells (Fig. 5c). These results reveal that METTL3-mediated m6A promotes *FOXO1* mRNA decay during cellular decidualization.

For further verification, we constructed a mutant (D395A and W398A, ovM3-MUT) of the *METTL3*-overexpressing vector with disordered enzymatic activity, as described previously [24]. We found that the mutant could elevate the expression of METTL3 similar to the wild-type *METTL3*-overexpressing (ovM3-WT) vector (Fig. 5d) but failed to elevate the m6A level of *FOXO1* mRNA in ThESCs compared with transfection with the ovM3-WT vector (Fig. 5e). In addition, the mRNA and protein levels of FOXO1 were decreased in ThESCs transfected with the ovM3-WT vector, but not in cells transfected with the ovM3-MUT vector (Fig. 5f,g). In addition, the decay rate of *FOXO1* mRNA accelerated rapidly in ThESCs transfected with the ovM3-WT vector but not in cells transfected with the ovM3-MUT vector in the transcription inhibition assay (Fig. 5h). These results indicate that METTL3-mediated m6A promotes the degradation of *FOXO1* mRNA.

Accumulating evidence suggests that YTHDF2 plays an important role in regulating RNA stability [28, 30]. Thus, RIP-PCR using an anti-YTHDF2 antibody was performed to test whether YTHDF2 is involved in the METTL3-mediated regulation of *FOXO1* mRNA degradation. The mRNA level of *FOXO1* bound to YTHDF2 increased in ThESCs transfected with the ovM3-WT vector but not in cells transfected with the ovM3-MUT vector (Fig. 5i), which is consistent with our previous results (Fig. 5e), suggesting the involvement of YTHDF2 in the METTL3-mediated regulation of *FOXO1* mRNA degradation. Taken together, we conclude that METTL3-mediated m6A promotes the degradation of *FOXO1* mRNA in a YTHDF2-dependent manner (Fig. 5j).

## Discussion

The findings of the present study indicate that METTL3-mediated m6A modification impairs the decidualization of endometrial stromal cells by promoting YTHDF2-dependent degradation of *FOXO1* mRNA, thus affecting embryo implantation and resulting in endometriosis-related infertility to a large extent. The main findings were as follows: (a) m6A and METTL3 levels in mid-secretory phase endometria were decreased compared to those in proliferative phase endometria in normal fertile women without endometriosis, while both m6A and METTL3 levels were elevated in the mid-secretory phase endometria of women with endometriosis-related infertility; (b) METTL3 expression gradually decreased during cellular decidualization, contrary to the expression of decidual markers, and METTL3 expression in decidual-treated ESCs derived from women with endometriosis-related infertility was higher than that in ESCs derived from normal fertile women; (c) increased METTL3 levels impaired the decidualization of ESCs by regulating the expression of FOXO1, thus affecting embryo implantation; and (d) METTL3-mediated m6A promoted the degradation of *FOXO1* mRNA in a YTHDF2-dependent manner.

It is widely recognized that m6A plays a crucial role in various biological and cellular processes [26–29]. Through the regulation of methyltransferases (writers), demethylases (erasers), and m6A-binding proteins (readers), m6A is dynamically and reversibly involved in these processes [15]. Recently, a growing body of evidence has demonstrated a connection between m6A and endometriosis. Down-regulated METTL3, heterogeneous nuclear ribonucleoprotein C (HNRNPC), and A2/B1 (HNRNPA2B1) have been linked to the development of endometriosis [19, 20]. Our prior research demonstrated that the suppression of METTL3 promoted the migration and invasion of endometrial stromal cells in endometriosis through the METTL3/m6A/miR126 axis [20]. Subsequent studies have provided similar results, indicating that reduced METTL3 stimulates the proliferation, invasion, and migration of endometrial stromal cells through m6A-mediated differential expression of downstream target genes, thus contributing to the development of endometriosis [31–33]. Additionally, two recent bioinformatics studies have identified several m6A regulators associated with endometriosis [34, 35]. One study reported that METTL3 and YTHDF2 were identified as potential diagnostic targets for endometriosis, suggesting the importance of the METTL3-m6A-mRNA/long non-coding RNA (lncRNA)-YTHDF2 axis in the development of endometriosis [34]. Our present study also supports the importance of this axis. Specifically, we have demonstrated the involvement of the METTL3-m6A-FOXO1-YTHDF2 axis in the decidualization of endometrial stromal cells. Another study has also identified three different m6A regulators (FTO, HNRNPC, and HNRNPA2B1) between the endometriosis and non-endometriosis groups [35]. Based on the analysis of these three candidate genes, the study identified three molecular subtypes, among which clusterB was found to be highly linked to endometriosis, with high levels of T helper 17 cells, neutrophil infiltration, and overexpression of pyroptosis-related genes [35]. Although numerous studies have emphasized the significance of m6A and its regulators in the pathogenesis of endometriosis, our understanding of m6A-associated molecular and cellular events in cellular decidualization and endometriosis-related infertility is still limited. Our present study demonstrated that m6A and METTL3 were upregulated in the mid-secretory phase of endometria from women with endometriosis-related infertility compared to those in fertile women without endometriosis, and that the upregulation of METTL3 impaired the decidualization of endometrial stromal cells, thus contributing to defective uterine receptivity and poor embryo implantation. While our previous studies as well as those of others have shown that decreased METTL3 is involved in the pathogenesis of endometriosis [20, 31–33]. Taken all together, METTL3 may have different functions in the pathogenesis and cellular decidualization of endometriosis.

Decidualization is a fundamental process in which endometrial fibroblast-like stromal cells undergo differentiation to form specialized decidual cells [25]. This process plays an important role in establishing endometrial receptivity, embryo implantation, and placental development, as it provides a vital nutritional and immunosuppressive substrate [25]. Unlike in most other mammals, decidualization in humans is triggered by an increase in progesterone and local cAMP secretion during the postovulatory phase of each menstrual cycle [25]. The transcription factor FOXO1 is activated by an increase in progesterone and cAMP levels in endometrial stromal cells, leading to cell cycle arrest and differentiation of stromal cells into decidual cells. These decidual cells are responsible for encasing and safeguarding the fetal/placental unit during gestation and offer specific endocrine and immune functions [36]. Therefore, FOXO1 is regarded as a vital marker for investigating cellular decidualization.

In this study, we investigated the effect of m6A on endometrial receptivity during normal physiological menstrual cycles and whether it is associated with defective endometrial receptivity in women with endometriosis-related infertility. To achieve this, we collected proliferative and mid-secretory endometria from normal fertile women and mid-secretory endometria from infertile women with endometriosis. Our results showed that m6A was expressed at a lower level in the mid-secretory phase of the normal endometrium than in the proliferative phase, suggesting that the downregulation of m6A may be associated with endometrial receptivity. We detected a series of m6A-related genes and found that METTL3, KIAA1429, and IGF2BPs were downregulated during the implantation window, whereas YTHDF3 was upregulated. Since METTL3 plays a core role in the m6A methyltransferase complex [12], we chose it for our in-depth study. Next, we detected the levels of m6A and METTL3 in the mid-secretory phase of the endometria of infertile women with endometriosis and found that both were expressed at higher levels in the mid-secretory endometria of these women than in those from normal fertile women. These results suggest that increased m6A/METTL3 expression is associated with defective endometrial receptivity in women with endometriosis. Additionally, we treated normal primary stromal cells with MPA and 8-Br-cAMP for in vitro decidualization and found that METTL3 expression decreased further with prolonged treatment time and was accompanied by reduced m6A levels. Various studies have shown that patients with endometriosis-related infertility have poor decidualization and reduced endometrial receptivity in the eutopic endometrium [9–11, 37–39]. Therefore, we treated two groups of primary stromal cells, one derived from women with endometriosis-infertility and the other from normal fertile women without endometriosis. We found that m6A and METTL3 levels were higher in eutopic stromal cells than in normal stromal cells, whereas FOXO1 levels were lower. These findings indicate that METTL3 negatively affects the cellular decidual process, contributing to defective endometrial receptivity in women with endometriosis. Furthermore, we demonstrated that *METTL3* overexpression suppressed the expression of decidualization markers, including FOXO1, PRL, and IGFBP1, and embryo implantation, whereas METTL3 knockdown had the opposite effect. Using RIP-PCR and MeRIP-PCR, we demonstrated that METTL3-mediated m6A promoted the decay of *FOXO1* mRNA in a YTHDF2-dependent manner. In summary, we revealed that METTL3-mediated m6A disrupts the cellular decidual process by promoting the YTHDF2-dependent decay of *FOXO1* mRNA, thereby contributing to defective endometrial receptivity in infertile women with endometriosis.

Additionally, the expression of IGF2BPs was notably lower in the mid-secretory phase endometria of normal fertile women than in the proliferative phase endometria. IGF2BP family members act as readers of m6A modification and are responsible for recognizing and binding the m6A signature, ultimately carrying out a variety of downstream functions. Previous studies have observed lower methylation ratios of IGF2BP2 in the endometrium on day 15 of gestation than on day 5, indicating that IGF2BP2 expression may be vital for embryo implantation [40]. IGF2BP3 controls embryonic development via alternative splicing of diverse genes [41]. During embryonic development in mice, IGF2BP1 and IGF2BP3 are primarily expressed in the snout, viscera, forebrain, hindbrain, branchial arch, skin, and tail vertebrae of mice [42, 43], whereas IGF2BP3 gradually degrades during the late embryogenesis stages, and IGF2BP1 remains in the kidney, intestine, and liver [44]. Conversely, IGF2BP2 is continuously expressed in many adult tissues [45, 46]. These findings reveal the essential roles of IGF2BPs in regulating embryonic development and suggest that their aberrant expression may play a role in tissue and organ dysplasia. Taken together, we propose that IGF2BPs may perform their functions in establishing endometrial receptivity, though more research is needed to provide further insights.

A recent study revealed the negative role of METTL3 in embryo implantation, which corroborates our viewpoint [47]. The study indicated a significant increase in global m6A methylation and METTL3 expression in the endometrial tissues of women with recurrent implantation failure compared to that in controls [47]. Overexpression of METTL3 in Ishikawa cells led to a decrease in the ratio of BeWo spheroid attachment and hindered the expression of homeobox A10 (HOXA10) and its downstream targets, whereas overexpression of HOXA10 in Ishikawa cells effectively restored the impact of METTL3 overexpression on embryo attachment in vitro [47]. Mechanistically, METTL3-mediated m6A contributed to the decay of HOXA10, thereby shortening its half-life [47]. Our study focused on the function of METTL3 in the decidualization of endometrial stromal cells and revealed a negative effect of METTL3 on the cellular decidual process in endometriosis, resulting in poor endometrial receptivity and disrupted embryo implantation. Interestingly, we found that the conclusions of two recent articles differed from our findings. Both of these studies [48, 49] used *Mettl3*-ablated mice and proposed a positive role for METTL3 in uterine receptivity and embryo implantation, as well as its crucial importance in the transmission of progesterone signals. Therefore, further studies are required to determine the effects of m6A/METTL3 on cellular decidualization, endometrial receptivity, and embryo implantation.

The uterine epithelium plays an important role in embryonic implantation. The uterine epithelium includes the luminal epithelium (LE) and glandular epithelium (GE), which extends from the LE to the stromal layer. The LE is the first maternal contact for an implanted embryo and serves as a transient gateway for embryo implantation and subsequent embryo development in the uterus [50]. During the implantation window, blastocyst attachment to the LE results in cellular and ultrastructural changes, including gradual loss of uterine epithelial cell polarity and formation of microprotrusions on the epithelial apical surface, which are called pinopodes or uterodomes [51, 52]. Efficient removal of the epithelial barrier by hatched blastocysts is a crucial step in embryo implantation [52]. Recent studies have shown that m6A plays an important role in epithelial development and differentiation. *Mettl3* deletion in the epidermis and oral epithelium results in broad developmental defects, including significant failure of hair morphogenesis, premature interfollicular differentiation, and loss of filiform papillae in the tongue [53]. The deletion of *Mettl14* in the murine epidermis impairs the m6A-dependent association between the long non-coding RNA plasmacytoma variant translocation 1 (Pvt1) and MYC, which is critical for the promotion of epidermal stemness and wound-healing capabilities [54]. These significant phenotypic abnormalities demonstrate the critical role of m6A in epithelial homeostasis. Increased METTL3-mediated m6A promotes the upregulation of p63 and K14, the downregulation of K10, and cell proliferation in cutaneous squamous cell carcinoma [55]. Increased m6A mediated by METTL3/METTL14 promotes tumorigenesis and tumor metastasis by regulating the stability and translation of oncogenic mRNA in several epithelial cancers [55–58]. These findings suggest a vital role for m6A in regulating the development, differentiation, and biological function of epithelial cells, which provides a direction for exploring endometrial receptivity and embryo implantation from the perspective of the association between m6A and the uterine epithelium.

## Conclusion

Our research reveals that m6A modification regulated by METTL3 impairs the decidual process of endometrial stromal cells by promoting YTHDF2-mediated degradation of *FOXO1* mRNA. This ultimately leads to poor uterine receptivity and embryo implantation failure. Our novel findings shed new light on the potential of METTL3 as a therapeutic target to enhance cellular decidual function in patients with endometriosis-related infertility and ultimately increase the probability of successful embryo implantation.

### Supplementary Information

**Additional file 1:** **Table S1.** The sequences of Primers. **Table S2.** The sequences of siRNAs. **Figure S1.** (a) Statistical analysis panel of FOXO1 levels in ESCs treated with the decidual treatment for 0 or 6 days. (b) Statistical analysis panel of METTL3 levels in ESCs treated with the decidual treatment for 0 or 6 days. (c) Dot blot assays were performed to detect m6A changes in ESCs derived from normal (ND) and endometriosis (ED) groups, both of which received decidual treatments. Methylene blue staining was used as a control. (d) Protein levels of FOXO1 in ESCs derived from ND and ED groups, both of which received decidual treatments. (e-h) mRNA levels of PRL and IGFBP1 upon *METTL3* overexpression or knockdown in ThESCs. All experiments were repeated in triplicate or quadruplicate. Data with error bars are presented to indicate the mean ± SEM values. *P < 0.05, **P < 0.01, ***P < 0.001. **Figure S2.** Statistical analysis panel of the FOXO1 levels in the epithelial cells on the DMSO-injected and STM2457-injected sides of the mouse uterus by IHC staining. All experiments were repeated in triplicate or quadruplicate. Data with error bars are presented to indicate the mean ± SEM values. *P < 0.05, **P < 0.01, ***P < 0.001.

**Additional file 2.**

## Data Availability

The data that support the findings of this study are available from the corresponding author upon reasonable request.

## Abbreviations

m6A: N6-methyladenosine
METTL3: Methyltransferase-like 3
WTAP: WT1 associated protein
VIRMA: Vir like m6A methyltransferase associated
ESCs: Primary endometrial stromal cells
DMEM/F12: Dulbecco’s Modified Eagle’s Medium
MPA: Medroxyprogesterone acetate
8-Br-cAMP: 8-Bromo-cyclic adenosine monophosphate
ThESCs: Human endometrial stromal cells
qRT-PCR: Quantitative real-time polymerase chain reactioN
FOXO1: Forkhead box O1
IHC: Immunohistochemical
IF: Immunofluorescence
PRL: Prolactin
IGFBP1: Insulin-like growth factor-binding protein 1
DMSO: Dimethyl sulfoxide
RIP-PCR: RNA immunoprecipitation PCR
MeRIP-PCR: Methylated RIP-PCR
ovM3-WT: Wild-type METTL3-overexpressing vector
ovM3-MUT: Mutated METTL3-overexpressing vector
Act D: Actinomycin D
HNRNPC: Heterogeneous nuclear ribonucleoprotein C
HNRNPA2B1: Heterogeneous nuclear ribonucleoprotein A2B1
lncRNA: Long non-coding RNA
HOXA10: Homeobox A10
LE: Luminal epithelium
GE: Glandular epithelium
Pvt1: Plasmacytoma variant translocation 1
