# Supplementary material for: METTL3-regulated m6A modification impairs the decidualization of endometrial stromal cells by regulating YTHDF2-mediated degradation of FOXO1 mRNA in endometriosis-related infertility
Source: Reprod Biol Endocrinol. 2023 Oct 27;21:99. doi: 10.1186/s12958-023-01151-0 (PMC10605339; doi:10.1186/s12958-023-01151-0)
Supplement: Supplementary file 1 — Additional file 1: Table S1. The sequences of Primers. Table S2. The sequences of siRNAs. Figure S1. (a) Statistical analysis panel of FOXO1 levels in ESCs treated with the decidual treatment for 0 or 6 days. (b) Statistical analysis panel of METTL3 levels in ESCs treated with the decidual treatment for 0 or 6 days. (c) Dot blot assays were performed to detect m6A changes in ESCs derived from normal (ND) and endometriosis (ED) groups, both of which received decidual treatments. Methylene blue staining was used as a control. (d) Protein levels of FOXO1 in ESCs derived from ND and ED groups, both of which received decidual treatments. (e-h) mRNA levels of PRL and IGFBP1 upon METTL3 overexpression or knockdown in ThESCs. All experiments were repeated in triplicate or quadruplicate. Data with error bars are presented to indicate the mean ± SEM values. *P < 0.05, **P < 0.01, ***P < 0.001. Figure S2. Statistical analysis panel of the FOXO1 levels in the epithelial cells on the DMSO-injected and STM2457-injected sides of the mouse uterus by IHC staining. All experiments were repeated in triplicate or quadruplicate. Data with error bars are presented to indicate the mean ± SEM values. *P < 0.05, **P < 0.01, ***P < 0.001. [file 12958_2023_1151_MOESM1_ESM.docx]

**Supplemental materials**

**Table S1. The sequences of Primers.**

| Names | Sequences |
| --- | --- |
| METTL3-F | CTATCTCCTGGCACTCGCAAGA |
| METTL3-R | GCTTGAACCGTGCAACCACATC |
| METTL14-F | CTGAAAGTGCCGACAGCATTGG |
| METTL14-R | CTCTCCTTCATCCAGATACTTACG |
| KIAA1429-F | TGACCTTGCCTCACCAACTGCA |
| LIAA1429-R | AGCAACCTGGTGGTTTGGCTAG |
| WTAP-F | GCAACAACAGCAGGAGTCTGCA |
| WTAP-R | CTGCTGGACTTGCTTGAGGTAC |
| ALKBH5-F | CCAGCTATGCTTCAGATCGCCT |
| ALKBH5-R | GGTTCTCTTCCTTGTCCATCTCC |
| FTO-F | CCAGAACCTGAGGAGAGAATGG |
| FTO-R | CGATGTCTGTGAGGTCAAACGG |
| YTHDF1-F | CAAGCACACAACCTCCATCTTCG |
| YTHDF1-R | GTAAGAAACTGGTTCGCCCTCAT |
| YTHDF2-F | TAGCCAGCTACAAGCACACCAC |
| YTHDF2-R | CAACCGTTGCTGCAGTCTGTGT |
| YTHDF3-F | GCTACTTTCAAGCATACCACCTC |
| YTHDF3-R | ACAGGACATCTTCATACGGTTATTG |
| IGF2BP1-F | CTTTGTAGGGCGTCTCATTGGC |
| IGF2BP1-R | CCTTCACAGTGATGGTCCTCTC |
| IGF2BP2-F | GTTGGTGCCATCATCGGAAAGG |
| IGF2BP2-R | TGGATGGTGACAGGCTTCTCTG |
| IGF2BP3-F | TCGTGACCAGACACCTGATGAG |
| IGF2BP3-R | GGTGCTGCTTTACCTGAGTCAG |
| FOXO1-F | CTACGAGTGGATGGTCAAGAGC |
| FOXO1-R | CCAGTTCCTTCATTCTGCACACG |
| IGFBP1-F | TTTTACCTGCCAAACTGCAACA |
| IGFBP1-R | CCCATTCCAAGGGTAGACGC |
| PRL-F | GGAGCAAGCCCAACAGATGAA |
| PRL-R | GGCTCATTCCAGGATCGCAAT |
| β-actin-F | CACCATTGGCAATGAGCGGTTC |
| β-actin-R | AGGTCTTTGCGGATGTCCACGT |
| m6A-FOXO1-F | CACCAGGAGAAGCTCCCAAG |
| m6A-FOXO1-R | TCCATCCATGAGGTCATTCCG |

**Table S2. The sequences of siRNAs.**

| Names | Sequences |
| --- | --- |
| si-NC-F | UUCUCCGAACGUGUCACGUTT |
| si-NC-R | ACGUGACACGUUCGGAGAATT |
| si-METTL3-F | GCAAGAAUUCUGUGACUAUTT |
| si-METTL3-R | AUAGUCACAGAAUUCUUGCAC |

Figure S1


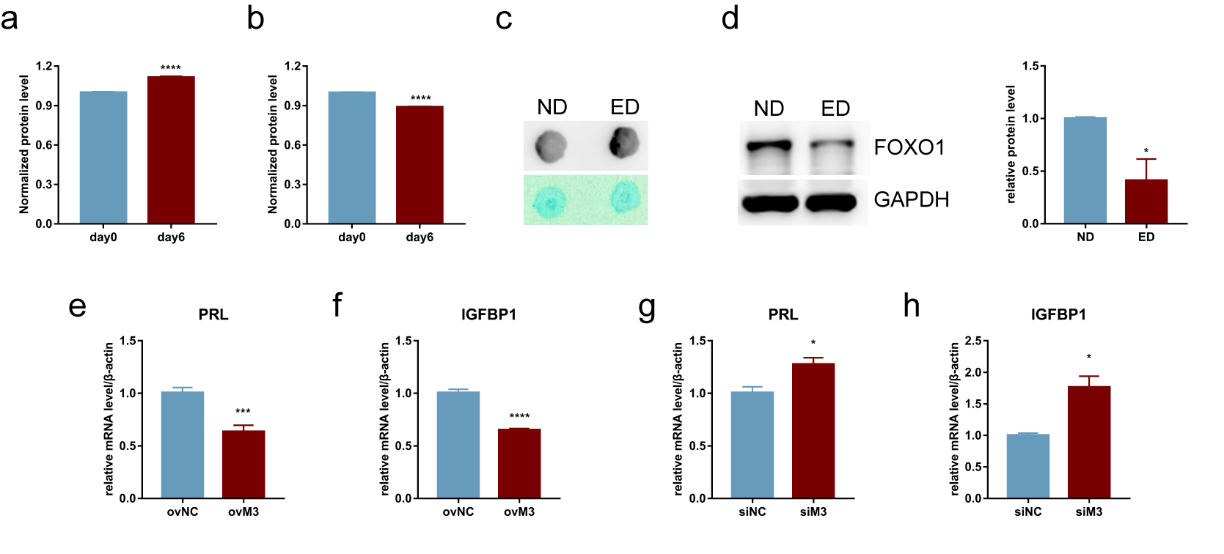


Figure S1. (a) Statistical analysis panel of FOXO1 levels in ESCs treated with the decidual treatment for 0 or 6 days. (b) Statistical analysis panel of METTL3 levels in ESCs treated with the decidual treatment for 0 or 6 days. (c) Dot blot assays were performed to detect m6A changes in ESCs derived from normal (ND) and endometriosis (ED) groups, both of which received decidual treatments. Methylene blue staining was used as a control. (d) Protein levels of FOXO1 in ESCs derived from ND and ED groups, both of which received decidual treatments. (e-h) mRNA levels of PRL and IGFBP1 upon *METTL3* overexpression or knockdown in ThESCs. All experiments were repeated in triplicate or quadruplicate. Data with error bars are presented to indicate the mean ± SEM values. *P < 0.05, **P < 0.01, ***P < 0.001.

Figure S2


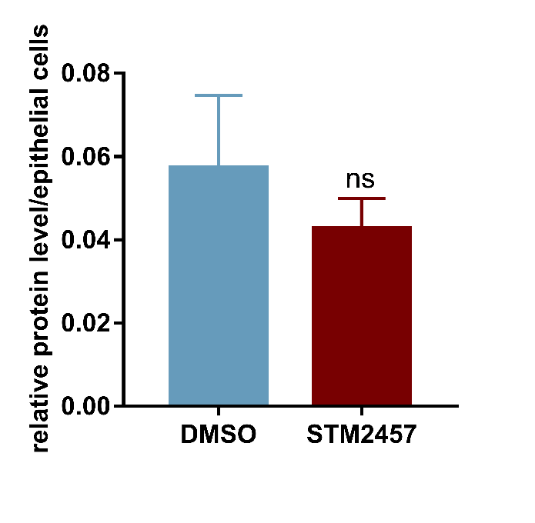


Figure S2.Statistical analysis panel of the FOXO1 levels in the epithelial cells on the DMSO-injected and STM2457-injected sides of the mouse uterus by IHC staining. All experiments were repeated in triplicate or quadruplicate. Data with error bars are presented to indicate the mean ± SEM values. *P < 0.05, **P < 0.01, ***P < 0.001.
