# Supplementary figures and images for: METTL3-regulated m6A modification impairs the decidualization of endometrial stromal cells by regulating YTHDF2-mediated degradation of FOXO1 mRNA in endometriosis-related infertility
Source: Reprod Biol Endocrinol. 2023 Oct 27;21:99. doi: 10.1186/s12958-023-01151-0 (PMC10605339; doi:10.1186/s12958-023-01151-0)

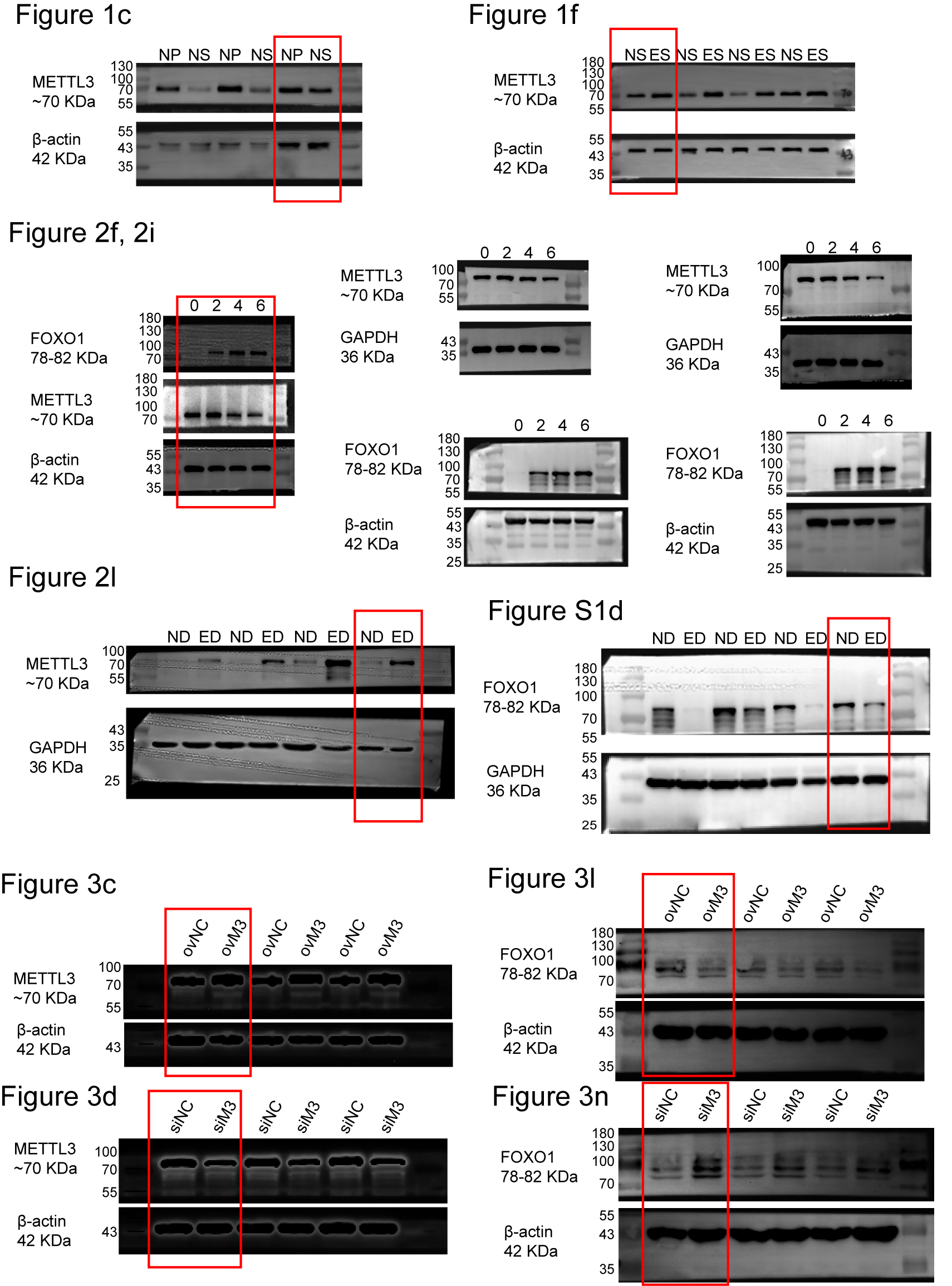

Supplement: Supplementary file 2 — Additional file 2. [file 12958_2023_1151_MOESM2_ESM.zip › 原始图1.tif]

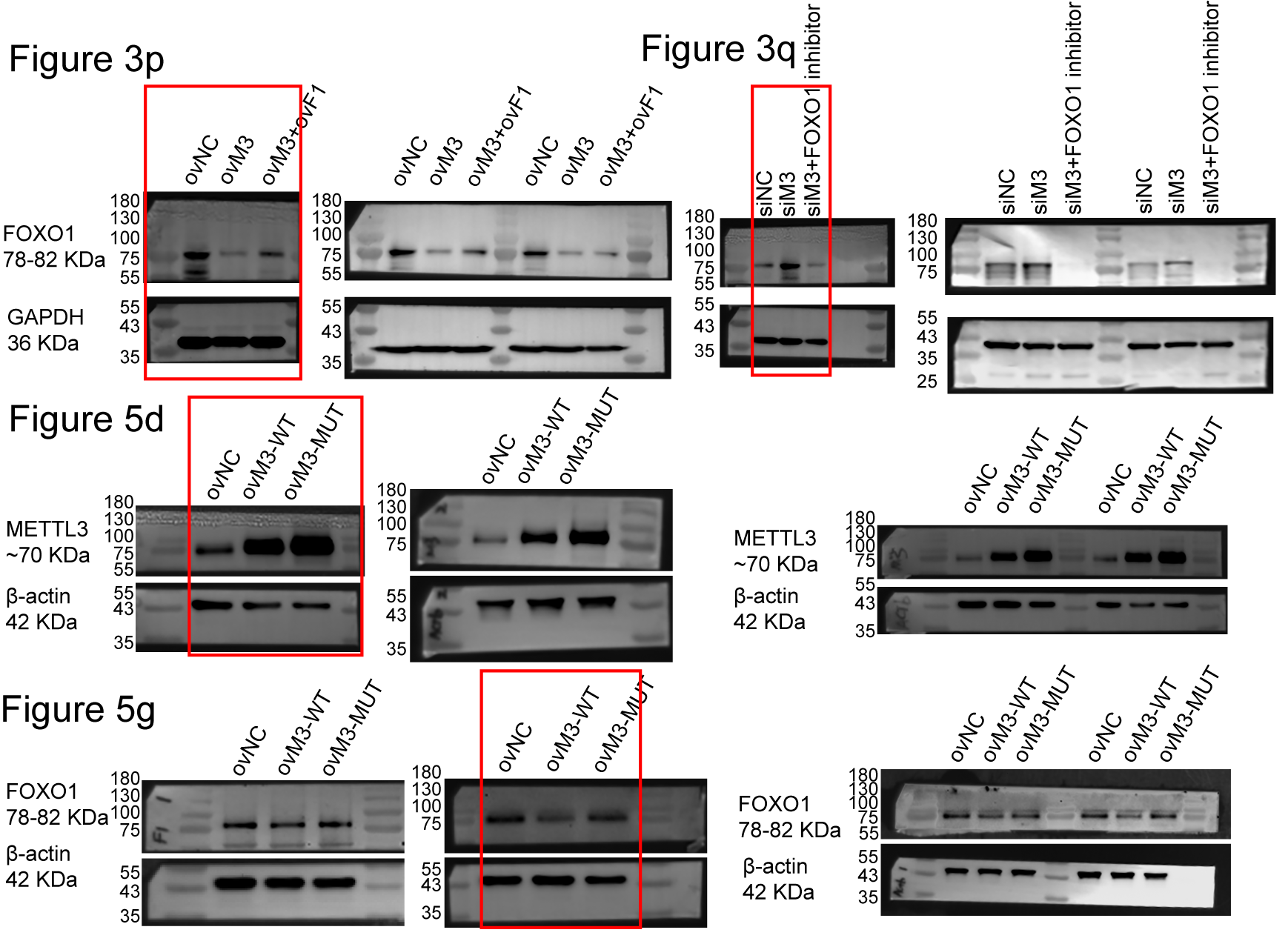

Supplement: Supplementary file 2 — Additional file 2. [file 12958_2023_1151_MOESM2_ESM.zip › 原始图2.tif]
